# Changes in Salivary Biomarkers and Oral Immune Parameters in Patients with Psoriasis: A Systematic Review

**DOI:** 10.3390/dj14030184

**Published:** 2026-03-19

**Authors:** Anna Skutnik-Radziszewska, Virginia Ewa Lis, Alicja Skutnik, Julita Szulimowska, Anna Zalewska

**Affiliations:** 1 Department of Conservative Dentistry, Medical University of Bialystok, 24A M. Sklodowskiej-Curie Street, 15-276 Bialystok, Poland; julita.szulimowska@umb.edu.pl (J.S.); anna.zalewska1@umb.edu.pl (A.Z.); 2 Doctoral School, Medical University of Bialystok, 1 Jana Kilinskiego Street, 15-089 Bialystok, Poland; virginia.lis@sd.umb.edu.pl; 3 Student Scientific Association at the Department of Conservative Dentistry, Medical University of Bialystok, 24A M. Sklodowskiej-Curie Street, 15-276 Bialystok, Poland; 45255@student.umb.edu.pl

**Keywords:** psoriasis, saliva, biomarkers, cytokines, oxidative stress

## Abstract

**Background:** Psoriasis is a chronic immune-mediated inflammatory disease characterized by systemic inflammation and complex immune dysregulation that extends beyond the skin and may affect the oral environment. Increasing evidence suggests that saliva may serve as a non-invasive diagnostic medium reflecting both local and systemic pathological processes. This systematic review aimed to critically evaluate current evidence on salivary biomarkers in psoriasis, focusing on inflammatory mediators, oxidative stress parameters, immune-related factors, and oral microbiota alterations, and to assess their potential clinical and diagnostic relevance. **Methods:** A systematic literature search was performed according to PRISMA guidelines using PubMed, Scopus, and Web of Science databases, covering studies published between 1994 and October 2024. Original human studies evaluating salivary biomarkers in patients with psoriasis were included based on predefined PECOS criteria. Studies involving confounding inflammatory oral diseases without separate analysis were excluded. Eleven eligible studies were included in a qualitative synthesis. **Results:** The analyzed studies consistently demonstrated multidimensional alterations in salivary composition in psoriasis patients compared with healthy controls. Increased levels of pro-inflammatory cytokines (TNF-α, IFN-γ, IL-2) and reduced anti-inflammatory IL-10 indicated persistent immune activation. Elevated oxidative stress markers, including total oxidant status and oxidative stress index, supported the role of redox imbalance in disease pathogenesis. Alterations in innate immune components, such as salivary α-amylase, immunoglobulin A, and lysozyme, suggested impaired oral immune regulation. Moreover, emerging microbiome data revealed shifts toward pro-inflammatory bacterial taxa, including Prevotella and Porphyromonas. Some studies indicated that biologic therapy may modulate salivary biomarker profiles. **Conclusions:** Salivary biomarkers reflect systemic inflammatory and immunological alterations associated with psoriasis and represent promising non-invasive tools for disease monitoring and clinical assessment. Nevertheless, substantial methodological heterogeneity and limited sample sizes highlight the need for large-scale, standardized, and longitudinal studies to validate their diagnostic applicability.

## 1. Introduction

### 1.1. Definition of Psoriasis

Psoriasis is a chronic, immune-mediated inflammatory skin disease. It is characterized by excessive proliferation of keratinocytes, hyperkeratinization, and increased angiogenesis, leading to the formation of typical erythematous, scaly plaques [1,2,3]. Psoriasis is estimated to affect between 0.3% and 2.5% of the global population, with a higher prevalence in high-income countries and in regions farther from the equator [4]. The most common clinical phenotype is plaque psoriasis (psoriasis vulgaris), accounting for approximately 90% of all cases [1,5]. In this form, patients develop characteristic, symmetrically distributed red lesions covered with silvery scales, most commonly affecting the scalp, lumbar region, and extremities [2,3].

Psoriasis is a systemic condition and, during exacerbations, may significantly reduce quality of life, causing both physical discomfort and psychological burden, including depression and anxiety disorders [6,7,8].

### 1.2. Etiology

Psoriasis is a multifactorial disease involving genetic, immunological, and environmental factors. Studies have shown that genetic predisposition plays an important role in disease development. In particular, the presence of the HLA-Cw6 allele is associated with increased susceptibility to plaque psoriasis [9,10,11]. Environmental factors, including trauma, infections, psychological stress, alcohol consumption, and tobacco use, may exacerbate psoriasis or trigger disease onset [9,12].

The pathophysiology of psoriasis involves immune system dysfunction, particularly excessive activation of T lymphocytes, dendritic cells, and macrophages [10,13]. These immune cells contribute to the production of numerous pro-inflammatory cytokines, including interleukins (IL-1β, IL-6), interferon gamma (IFN-γ), and tumor necrosis factor alpha (TNF-α) [14,15,16]. Cytokines associated with the T helper 1 (Th1) and T helper 17 (Th17) immune profiles, such as IL-17 and TNF-α, play a key role in amplifying cutaneous inflammation. This process leads to uncontrolled keratinocyte proliferation and increased migration of immune cells into the skin [17,18].

Excessive oxidative activity is another important factor in the pathogenesis of psoriasis and contributes to cellular damage and intensification of inflammation [19,20]. Reactive oxygen species (ROS) induce cellular injury, lipid peroxidation, and protein modifications, thereby aggravating inflammation and exacerbating psoriasis symptoms [19,20,21,22]. Therefore, oxidative stress biomarkers may represent promising candidates for diagnostic and prognostic assessment, as discussed later in this paper.

### 1.3. Associations with Other Diseases

As a systemic disease, psoriasis is strongly associated with a range of other conditions. As an autoimmune-related disorder, it frequently co-occurs with other autoimmune diseases, suggesting shared pathogenic mechanisms. Studies have shown that patients with psoriasis have an increased risk of developing type 2 diabetes, metabolic syndrome, and chronic kidney disease [23,24,25,26,27]. One of the most commonly coexisting craniofacial conditions in patients with psoriasis is periodontitis. Epidemiological studies indicate that periodontitis may increase the risk of early-onset psoriasis [28,29]. Conversely, individuals with psoriasis have a higher risk of developing periodontitis. The severity of periodontal disease is also significantly greater compared to healthy individuals [30]. Dysbiosis in the periodontal pocket is considered a key factor in this bidirectional relationship, which contributes to the chronic inflammatory state. It promotes the production of interleukins [31,32,33,34], prostaglandins [35], and leukotrienes [36], activates osteoclasts [37], leads to bone destruction, and facilitates the translocation of oral bacteria into the systemic circulation [38]. The systemic inflammatory nature of psoriasis is characterized by persistent immune activation, increased cytokine production, and enhanced oxidative stress [39]. As a result, disease-related molecular changes are not limited to the skin but can also be detected in various body fluids [40]. Circulating inflammatory mediators, oxidative stress markers, and immune-related proteins may enter exocrine secretions, including saliva [41]. Therefore, the analysis of salivary biomarkers may reflect not only local oral conditions but also systemic pathological processes associated with psoriasis. This supports the use of saliva as a non-invasive source of biomarkers for monitoring disease activity.

### 1.4. Saliva as a Diagnostic Material in Psoriasis

Saliva, as a biological fluid, has gained increasing interest in the diagnosis of various inflammatory diseases [41,42]. It contains numerous components, such as enzymes, proteins, cytokines, and antioxidants, which reflect the physiological and pathological state of the body and may serve as biomarkers of different disease processes [43,44,45]. The literature describes the use of saliva in the diagnosis of diabetes, chronic heart and kidney diseases, autoimmune disorders, as well as in monitoring the abuse of psychoactive substances [46,47,48,49,50]. Due to its ease of collection, non-invasiveness, and low cost, saliva is increasingly used as a diagnostic material and a potential alternative to blood testing, making it a practical tool for clinicians [51,52].

### 1.5. Objectives and Rationale

The objective of this study was to systematically review research on the use of saliva as a diagnostic material in psoriasis, with particular emphasis on oxidative stress and inflammatory biomarkers. This review analyzes changes in salivary biomarker levels in patients with psoriasis and the analytical methods used for their detection.

Unlike other reviews that include patients with accompanying periodontitis, the present analysis focuses exclusively on patients diagnosed with psoriasis [53,54,55]. This approach is important because other chronic inflammatory diseases, especially periodontal disease, may significantly influence salivary biomarker levels and act as confounding factors. By limiting the analysis to psoriasis alone, a more objective assessment of the diagnostic and monitoring potential of salivary biomarkers can be achieved.

This review also evaluates the usefulness of saliva as a non-invasive diagnostic tool for monitoring disease progression, assessing disease activity, and identifying potential systemic complications in patients with psoriasis.

## 2. Materials and Methods

### 2.1. Search Strategy and Data Extraction

This systematic review was conducted and reported in accordance with the Preferred Reporting Items for Systematic Reviews and Meta-Analyses (PRISMA 2020) guidelines [56]. The completed PRISMA 2020 checklist is provided in the Supplementary Materials. The methodological approach of the review was additionally guided by the recommendations of the Joanna Briggs Institute (JBI) for systematic reviews of observational studies. A systematic literature search was performed from 1994 to October 2024. The literature search was conducted in the following databases: PubMed, Scopus, and Web of Science. The detailed search strategy was developed a priori to ensure reproducibility. For each database, controlled vocabulary (where applicable) and free-text terms related to psoriasis, saliva, and salivary biomarkers were combined using Boolean operators (AND, OR). The following example search string was used in PubMed: (“Psoriasis”[Mesh] OR psoriasis[tiab]) AND (“Saliva”[Mesh] OR saliva*[tiab] OR salivary[tiab]) AND (“Oxidative Stress”[Mesh] OR oxidat*[tiab] OR antioxidant*[tiab] OR “Inflammation”[Mesh] OR inflammat*[tiab] OR cytokine*[tiab] OR biomarker*[tiab] OR proteomic*[tiab] OR “Microbiota”[Mesh] OR microbiot*[tiab] OR microbial[tiab]). For Scopus, the search was conducted in titles, abstracts, and keywords using the following string: TITLE-ABS-KEY (psoriasis AND (saliva* OR salivary) AND (“oxidative stress” OR oxidative OR antioxidant* OR inflammation OR cytokine* OR biomarker* OR proteomic* OR microbiot*)). For Web of Science, an analogous strategy was applied: TS = (psoriasis AND (saliva* OR salivary) AND (“oxidative stress” OR oxidative OR antioxidant* OR inflammation OR cytokine* OR biomarker* OR proteomic* OR microbiot*)).

**Figure 1:**
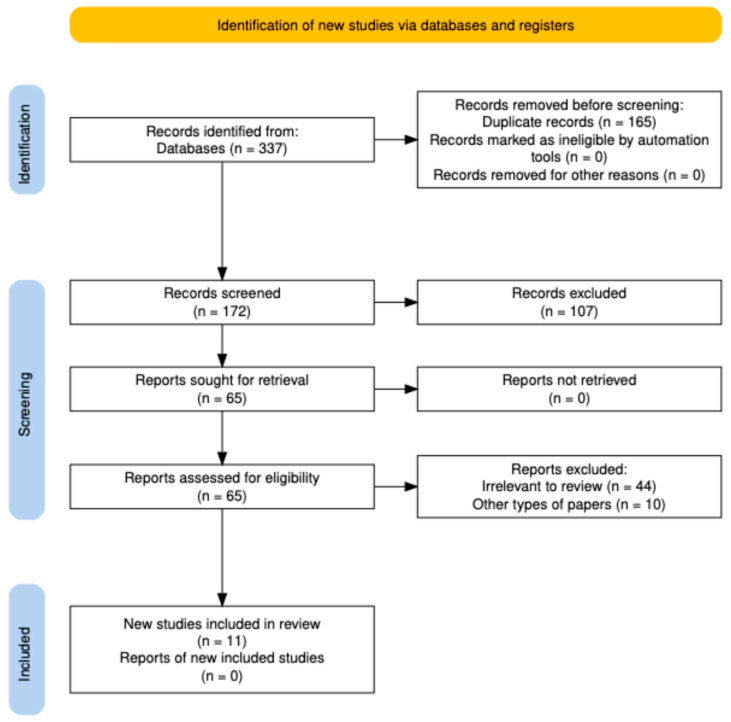
PRISMA flow diagram of the study selection process. Records removed before screening (duplicate records removed). Full-text articles excluded after eligibility assessment with reasons.

Duplicate and overlapping records were manually removed using Zotero reference management software (Zotero, version 6.0, Corporation for Digital Scholarship, Vienna, VA, USA) by one reviewer (A.S.-R.). Records were screened based on title, abstract, and full text by two independent reviewers (A.S.-R. and V.L.). Data extraction was performed independently by two reviewers (A.S.-R. and V.L.) using a standardized data extraction form developed prior to the review. The following data were extracted from each included study: study design, sample size, participant characteristics, type of saliva analyzed (stimulated or non-stimulated), saliva collection protocol, analytical methods, assessed biomarkers, and main outcomes. Any discrepancies between reviewers were resolved through discussion and, if necessary, consultation with a third reviewer (A.Z.). Studies were included if they fully met the predefined criteria based on the PECOS framework (Population, Exposure, Comparison, Outcomes, Study Design), as detailed in Table 1. A detailed flow diagram of the search process is presented in Figure 1.

The PECOS criteria were defined as follows:

Population (P): human subjects diagnosed with psoriasis, regardless of age, sex, or disease severity. Exposure (E): assessment of salivary biomarkers, including oxidative stress markers, inflammatory mediators, proteins, antioxidants, or microbiological parameters. Comparator (C): healthy control subjects without psoriasis or, where applicable, intra-group comparisons based on disease activity or severity. Outcomes (O): quantitative or qualitative changes in salivary biomarker levels and their association with the presence, activity, or severity of psoriasis. Study Design (S): original observational studies, including cross-sectional, case–control, and cohort studies [57].

Studies were excluded if they: (1) included patients with coexisting chronic inflammatory oral diseases (e.g., periodontitis) without separate subgroup analysis for psoriasis; (2) were case reports, narrative reviews, conference abstracts, or animal studies; (3) lacked original data on salivary biomarkers; or (4) were not published in English. Research letters were eligible for inclusion only if they contained original quantitative data.

Given the long search period (1994–2024), analytical techniques used for salivary biomarker assessment have evolved substantially over time. Earlier studies predominantly relied on conventional methods such as spectrophotometric assays and single-analyte enzyme-linked immunosorbent assays (ELISA), whereas more recent investigations increasingly employ high-sensitivity and high-throughput techniques, including multiplex immunoassays, mass spectrometry, and molecular-based analytical methods. These methodological advancements have significantly improved the sensitivity, specificity, and reliability of salivary biomarker measurements, which were taken into account during data interpretation.

Quantitative synthesis (meta-analysis) was not performed due to substantial heterogeneity among the included studies. The heterogeneity concerned the types of salivary biomarkers analyzed (oxidative stress markers, cytokines, proteins, and microbiological parameters), laboratory methodologies, assay platforms, study populations, clinical severity scales, and outcome reporting formats. This level of variability precluded meaningful statistical pooling of the results and therefore justified the use of a qualitative systematic synthesis.

### 2.2. Registration

This systematic review was registered in the OSF Registries (Open Science Framework) under the identifier DJSQM (https://doi.org/10.17605/OSF.IO/DJSQM), accessed on 26 November 2025. The full review protocol, including the eligibility criteria, search strategy, and data extraction plan, is openly accessible at the registration website. No amendments to the protocol were introduced after registration. Minor editorial clarifications were made that did not affect the predefined objectives, inclusion/exclusion criteria, or analysis strategy.

### 2.3. Quality Assessment and Critical Appraisal for the Systematic Review of Included Studies

The risk of bias in each included study was assessed using the quality assessment tools developed by the National Heart, Lung, and Blood Institute [58]. The NIH assessment tools evaluate key methodological domains, including clarity of the research question, adequacy of the study population definition, participation rate, sample size justification, validity and reliability of exposure and outcome measurements, consistency of outcome assessment, blinding of outcome assessors, and control for potential confounding variables. Each criterion is rated as “yes,” “no,” “cannot determine,” “not applicable,” or “not reported.” For quantitative quality summarization, each study was assigned a numerical score (1 point for low risk of bias, 0.5 for unclear risk, and 0 for high risk). Based on the total score, studies were classified as having good, intermediate, or poor quality. The results of the quality assessment were interpreted primarily at the domain level rather than relying solely on the aggregated numerical score. The overall level of evidence was additionally determined according to the Oxford Centre for Evidence-Based Medicine (OCEBM) classification for diagnostic studies. This system ranks evidence from level 1 (highest) to level 5 (lowest) based on study design and methodological rigor.

The checklists were completed independently by two reviewers (A.S.-R., A.Z.), and any discrepancies were resolved through discussion. The quality assessment results for individual studies are presented in Table 2. The most frequently identified methodological limitations included the lack of information regarding blinding and the absence of sample size justification, which were observed in several studies. Because the majority of included studies were observational case–control designs, randomization was not expected and therefore was not interpreted as a major methodological limitation. Seven studies were rated as having good quality, while four were considered of intermediate quality. All included studies were classified as level three or four on the five-level Oxford Centre for Evidence-Based Medicine scale [59].

## 3. Results

### 3.1. Study Selection

The database search identified a total of 337 records across PubMed, Scopus, and Web of Science. After removing duplicates, 172 records remained for title and abstract screening. During this stage, 107 articles were excluded because they did not meet the inclusion criteria. The full texts of 65 studies were subsequently assessed for eligibility. Of these, 54 were excluded due to reasons such as lack of salivary biomarker data, absence of a psoriasis study group, non-original study design, or insufficient methodological information. Finally, 11 studies met the predefined eligibility criteria and were included in the qualitative synthesis. The detailed process of study identification, screening, eligibility assessment, and inclusion is presented in Figure 1. The most common reasons for exclusion at the full-text stage included lack of salivary biomarker data, absence of a psoriasis study group, non-original study design, or insufficient methodological information.

### 3.2. Study Characteristics

A total of 11 studies published between 2004 and 2024 were included in the systematic review. The characteristics of the included studies are summarized in Table 3. The studies evaluated a wide range of salivary biomarkers in patients with psoriasis, including pro- and anti-inflammatory cytokines, oxidative and nitrosative stress markers, immune defense proteins, neuroendocrine mediators, and components of the oral microbiota. The sample sizes of the included studies varied considerably, and most investigations used case–control designs comparing patients with psoriasis to healthy control groups.

The saliva analyzed in the studies included both non-stimulated whole saliva (NWS) and stimulated whole saliva (SWS). Detailed pre-analytical characteristics of saliva sampling protocols used in the included studies are presented in Supplementary Table S1. Different analytical techniques were used across the studies, including enzyme-linked immunosorbent assay (ELISA), spectrophotometry, electrochemiluminescence immunoassay (ECLIA), and high-throughput sequencing approaches for microbiota analysis.

### 3.3. Risk of Bias in Included Studies

The methodological quality of the included studies was assessed using the National Institutes of Health (NIH) Quality Assessment Tool for Observational Cohort and Cross-Sectional Studies. The results of the risk-of-bias assessment are presented in Table 2.

Overall, most studies demonstrated intermediate methodological quality. The most common methodological limitations included relatively small sample sizes, limited reporting of saliva collection standardization, and insufficient control for potential confounding variables such as oral health status, systemic comorbidities, or medication use. However, most studies clearly defined the study population and applied appropriate laboratory methods for biomarker detection.

### 3.4. Results of Individual Studies

The included studies investigated a diverse set of salivary biomarkers associated with inflammatory processes, oxidative stress, immune defense mechanisms, neuroendocrine activity, and microbial composition.

Several studies reported elevated concentrations of pro-inflammatory cytokines in the saliva of patients with psoriasis, including tumor necrosis factor alpha (TNF-α), interferon gamma (IFN-γ), interleukin-2 (IL-2), and interleukin-1β (IL-1β). In contrast, decreased levels of the anti-inflammatory cytokine interleukin-10 (IL-10) were reported in some studies.

Markers of oxidative stress were also frequently altered in patients with psoriasis. Increased levels of total oxidant status (TOS), oxidative stress index (OSI), and lipid peroxidation products were reported, suggesting the presence of redox imbalance in saliva.

Changes in immune defense proteins were observed as well. Some studies reported reduced concentrations of secretory immunoglobulin A (sIgA) and lysozyme, which may indicate impaired local immune defense mechanisms within the oral cavity.

Neuroendocrine markers such as salivary α-amylase, cortisol, and chromogranin A were also evaluated. These markers were suggested to reflect activation of stress-related neuroendocrine pathways potentially associated with inflammatory disease activity.

Recent studies applying high-throughput sequencing approaches also identified alterations in the composition of the oral microbiota in patients with psoriasis. Increased relative abundance of certain bacterial genera, including Alloprevotella and Porphyromonas, was reported, suggesting potential associations between microbial dysbiosis and inflammatory processes.

### 3.5. Synthesis of Results

Across the included studies, a consistent pattern emerged indicating that psoriasis is associated with multidimensional alterations in salivary composition. These changes include increased levels of pro-inflammatory cytokines, elevated oxidative stress markers, alterations in immune defense proteins, changes in neuroendocrine mediators, and shifts in oral microbial communities.

Taken together, the available evidence suggests that saliva reflects both local oral immune activity and systemic inflammatory processes associated with psoriasis. These findings highlight the potential of salivary biomarkers as non-invasive indicators of disease activity and inflammatory status in patients with psoriasis.

Due to the heterogeneity of study designs, biomarker panels, and analytical methods, a quantitative meta-analysis was not performed. Instead, a qualitative synthesis of the findings was conducted. Due to substantial heterogeneity in study designs, biomarker panels, and analytical methodologies, a quantitative meta-analysis was not performed.

### 3.6. Reporting Bias

No formal assessment of reporting bias was performed due to the limited number of included studies and the heterogeneity of reported outcomes. The detailed risk-of-bias assessments for each individual study are presented in Table 2.

## 4. Discussion

### 4.1. Dysfunctions of Innate and Adaptive Immune Mechanisms—Salivary Enzymes and Their Role in the Pathogenesis of Psoriasis

Innate and adaptive salivary immunity plays a crucial role in maintaining oral homeostasis and protection against pathogens. Innate immunity is mediated by antimicrobial proteins such as lysozyme, lactoferrin, defensins, and histatins, which inhibit microbial growth and adhesion through direct bactericidal activity, iron sequestration, and membrane disruption. Adaptive immunity, primarily dependent on sIgA, neutralizes pathogens and prevents their adhesion to mucous membranes by immune exclusion and inhibition of microbial epithelial attachment, providing protection against bacterial, viral, and fungal infections [70,71,72].

Soudan et al. demonstrated increased salivary amylase activity in non-stimulated whole saliva (NWS) of patients with psoriasis compared to healthy individuals [67]. This finding is consistent with observations by Syrjanen, who reported a significant increase in this enzyme’s activity in parotid saliva of psoriatic patients [73]. The authors suggested that elevated salivary amylase activity may result from beta-adrenergic system stimulation and may reflect the emotional state of individuals with psoriasis. At the molecular level, β-adrenergic receptor activation in salivary acinar cells leads to increased intracellular cyclic adenosine monophosphate (cAMP) signaling, which directly enhances α-amylase gene transcription and secretion. These conclusions were supported by studies by Inagaki et al. and Kivlighan and Granger [74,75], which indicated a predominant role of the sympathetic nervous system and a marginal role of the parasympathetic system in amylase secretion under psychosocial stress. Findings on other components of salivary immunity, including sIgA and lysozyme, are inconsistent, possibly due to variations in study group sizes and the lack of standardized saliva collection protocols. Due to the time frame of this review, we cite the results of Koh et al. [69]. These authors observed significantly lower concentrations of sIgA and lysozyme in the saliva of psoriatic patients compared to controls; however, in NWS, sIgA levels were only slightly lower and lysozyme levels slightly higher in patients with more severe psoriasis than in those with moderate disease severity [69]. A reduction in the concentration or activity of immune components may be associated with decreased mucosal immunity in the oral cavity and increased susceptibility to bacterial infections. Mechanistically, reduced sIgA impairs immune exclusion at the mucosal surface, while decreased lysozyme limits bacterial cell wall degradation, facilitating oral microbial overgrowth and low-grade mucosal inflammation. An interesting observation from both studies (Soudan et al. and Koh et al.) is the lack of correlation between the activity/concentration of the examined parameters and the Psoriasis Area and Severity Index (PASI) score or disease duration [67,69].

Recent narrative reviews have emphasized that oral fluids, including saliva and gingival crevicular fluid, are promising non-invasive sources of diagnostic and monitoring biomarkers in psoriasis, further supporting the rationale for salivary biomarker research in this disease [76].

### 4.2. Oxidative and Nitrosative Stress Biomarkers in Psoriasis

Oxidative stress plays a significant role in the pathogenesis of psoriasis, leading to an imbalance between ROS and antioxidant defense mechanisms. In patients with psoriasis, significantly elevated levels of salivary antioxidant enzymes such as catalase (CAT), peroxidase (Px), and superoxide dismutase (SOD) have been observed, suggesting a compensatory upregulation of endogenous antioxidant defense systems in response to excessive reactive oxygen species generation [42]. This finding supports the systemic nature of psoriasis and highlights the role of redox imbalance in activating pro-inflammatory signaling pathways, sustaining chronic inflammation, promoting keratinocyte hyperproliferation, and potentially contributing to endothelial dysfunction and vascular complications.

Skutnik-Radziszewska et al. reported significantly increased TOS and OSI levels in the saliva of psoriasis patients compared to healthy controls [42]. A similar plasma redox imbalance shifting toward oxidation has been confirmed by other researchers investigating plaque psoriasis [77,78]. The authors suggest that the positive correlation between salivary levels of TOS, OSI, and lipid hydroperoxides (LOOH) and psoriasis severity indicates that oxidative stress in the oral cavity may exacerbate inflammatory processes in psoriasis through lipid peroxidation, membrane damage, and amplification of cytokine-driven signaling cascades. Furthermore, Skutnik-Radziszewska et al. point to the potential role of TOS and OSI as diagnostic and prognostic biomarkers [42]. Their reported sensitivity and specificity in differentiating psoriatic patients from healthy individuals may indicate potential usefulness in monitoring disease progression and assessing treatment efficacy.

Elevated levels of salivary nitric oxide (NO) in psoriasis patients result from enhanced nitrosative stress associated with chronic inflammation. Studies have shown significantly elevated concentrations of NO and nitrotyrosine in both NWS and stimulated whole saliva (SWS) samples of psoriatic patients, with these increases correlating with disease duration and severity of skin lesions as measured by the PASI [41]. Additionally, the overproduction of NO is driven by the activation of inducible nitric oxide synthase (iNOS) in response to pro-inflammatory cytokines (IL-2, TNF-α), which may contribute to nitrosative modification of proteins, mitochondrial dysfunction, structural damage of salivary glands, reduced secretory function, and subsequent exacerbation of psoriatic symptoms [79,80].

### 4.3. Pro-Inflammatory Cytokines in Saliva as a Reflection of Chronic Inflammation and Psoriasis Progression

Evidence indicates that the activation of phospholipase A2 (PLA2) leads to the release of arachidonic acid (AA) from cell membranes, a key component of inflammatory signaling pathways. AA is subsequently metabolized by cyclooxygenases (COX) and lipoxygenases (LOX) into eicosanoids, such as prostaglandins, leukotrienes, and thromboxanes. These lipid mediators amplify inflammatory responses in the skin through activation of nuclear factor κB (NF-κB), mitogen-activated protein kinase (MAPK) pathways, and downstream transcription of pro-inflammatory genes. In the context of psoriasis and other chronic inflammatory diseases, the overactivation of these signaling pathways promotes increased production of pro-inflammatory cytokines (e.g., IL-1, IL-6, TNF-α), through sustained activation of immune effector cells, enhanced leukocyte recruitment, and stimulation of keratinocyte proliferation and aberrant differentiation. 

Chronic inflammation in psoriasis is reflected in altered salivary cytokine profiles. Studies by Sharma et al. and Skutnik-Radziszewska et al. demonstrated significantly elevated levels of TNF-α, IFN-γ, and IL-2 in patients with psoriasis compared to healthy controls, alongside a concurrent reduction in IL-10 levels [41,61,62,63]. This imbalance between pro- and anti-inflammatory cytokines indicates a disruption in immune regulation that may contribute to the persistence of inflammation in the salivary glands through continuous cytokine-driven immune cell infiltration and local tissue remodeling.

Moreover, a positive correlation between the levels of TNF-α, IL-2, and IFN-γ and disease severity as measured by the PASI suggests their potential role as biomarkers of disease progression [41]. Conversely, the absence of a significant relationship between IL-10 levels and PASI indicates that this cytokine may have limited prognostic value for psoriasis severity, despite its known regulatory function in immune responses [61]. Skutnik-Radziszewska et al. propose that the reduced salivary flow in psoriatic patients, correlated with elevated levels of pro-inflammatory cytokines, may result from chronic inflammation affecting salivary gland function through cytokine-mediated microvascular alterations, acinar cell dysfunction, and impaired secretory signaling [41]. This could promote dysbiosis of the oral microbiota and increase the risk of secondary bacterial and fungal infections—factors that may further aggravate inflammation and contribute to disease progression.

The elevated salivary IL-1β levels observed in psoriatic patients compared to controls suggest that production of this cytokine is upregulated due to the chronic inflammatory state associated with psoriasis [54,66,68,81]. The phenomenon of no further increase in IL-1β in response to stress (the so-called “ceiling effect”) may indicate dysregulation of the neuroimmune axis in psoriasis, possibly due to persistently elevated IL-1β levels leading to desensitization of stress-responsive signaling pathways and impaired adaptive neuroimmune responses [68]. This altered regulation may contribute to the maintenance of chronic cutaneous inflammation and exacerbation of psoriatic symptoms.

These observations are in line with recent reviews on systemic biomarkers of psoriasis severity, which identify inflammatory and oxidative stress markers as particularly promising candidates for quantifying disease activity and treatment response through direct reflection of immune activation and redox-dependent signaling pathways [82].

### 4.4. The Impact of Biologic Therapy on Salivary Biomarkers—A Potential Tool for Monitoring Treatment Efficacy

Biologic therapy is a key strategy for managing severe psoriasis, and its effects on salivary biomarkers may provide valuable insight into treatment efficacy and disease pathophysiology through targeted inhibition of key inflammatory signaling pathways, including TNF-α-, IL-17-, and IL-23-dependent cascades. Findings from Foks-Ciekalska et al. and Ganzetti et al. demonstrated significant changes in salivary biomarker levels, including sIgA, salivary alpha-amylase (sAA), chromogranin A (CgA), and IL-1β, in response to biologic treatment [64,81].

Foks-Ciekalska et al. reported that biologic therapy was associated with an increase in sIgA and sAA concentrations in NWS of patients with psoriasis, compared to pre-treatment levels. This may indicate improved mucosal immune function and enhanced oral defense mechanisms as a result of reduced cytokine-mediated epithelial barrier disruption and restored immune homeostasis [64]. The post-treatment increase in sIgA may reflect improved immune status and decreased susceptibility to infections, consistent with previous research on the mechanisms of biologic therapy in autoimmune diseases [83,84,85]. Meanwhile, the rise in sAA during treatment may reflect adaptive modulation of sympathetic nervous system activity and its role in regulating salivary gland secretory signaling via β-adrenergic receptor-dependent pathways. A similar phenomenon has been observed in other autoimmune conditions, such as Sjögren’s syndrome [85,86,87].

An interesting observation is the concurrent decrease in CgA levels after biologic therapy, suggesting a reduction in psychosocial stress in psoriatic patients [64]. CgA is considered a marker of hypothalamic–pituitary–adrenal (HPA) axis activation. Its decline following treatment may indicate reduced stress and improved psychophysical well-being through normalization of hypothalamic–pituitary–adrenal (HPA) axis activity and attenuation of stress-induced immune activation [88,89]. Similar patterns have been reported in inflammatory bowel diseases (IBD), where elevated CgA levels were linked to active inflammation and responded to biologic therapy [90].

Ganzetti et al. observed a significant reduction in IL-1β levels in the saliva of patients treated with TNF-α inhibitors, suggesting that biologic therapy may contribute to the alleviation of inflammation at the level of the salivary glands through direct suppression of TNF-α–driven inflammatory cascades and downstream IL-1β production [66,81]. Comparable findings were obtained in studies on the use of anti-TNF therapy in ulcerative colitis and Crohn’s disease, where reductions in IL-1β levels correlated with clinical improvement and disease activity in colonic tissue [90,91].

Additionally, a correlation was found between IL-1β salivary levels and the severity of psoriatic skin lesions, as measured by PASI, after biologic treatment. This finding supports IL-1β as a potentially sensitive candidate biomarker of treatment efficacy in psoriasis [66,81]. The absence of such a correlation prior to treatment suggests that the observed reduction in IL-1β may result directly from the immunomodulatory effects of biologic therapy mediated by targeted cytokine neutralization and downstream suppression of inflammatory gene transcription.

### 4.5. Oral Microbiota Alterations in Psoriasis Patients—New Perspectives in Disease Pathogenesis Research

Zhao et al. demonstrated significantly higher alpha diversity of the oral microbiota in patients with psoriasis compared to healthy controls, suggesting greater richness and evenness of microbial composition [92,93]. These findings are consistent with previous reports of microbiota alterations in other inflammatory diseases such as rheumatoid arthritis and systemic lupus erythematosus [94]. In contrast, Belstrøm et al. found no significant differences in alpha diversity between groups, a discrepancy potentially due to different sequencing methodologies, population characteristics, and racial backgrounds [95]. At the same time, beta diversity analysis revealed significant differences in bacterial community structure between psoriasis patients and healthy individuals, potentially reflecting different immune activity in psoriasis [60]. Recent systematic reviews of oral and gut microbiota in psoriatic disease have similarly reported that patients with psoriasis tend to exhibit distinct microbial profiles compared with healthy controls. However, the available results are heterogeneous and largely based on small observational studies with variable methodology [96]. At the molecular level, these microbial shifts are thought to influence psoriasis through modulation of innate immune signaling, particularly via Toll-like receptor (TLR) activation and downstream NF-κB-dependent cytokine transcription.

Similar findings have been reported for other anatomical sites. A recent study of the scalp microbiome in psoriasis demonstrated increased bacterial diversity and a higher relative abundance of Pseudomonas in patients with severe scalp involvement compared with those with mild disease, further supporting the concept that site-specific dysbiosis may be linked to psoriasis severity [97].

The presence of *Alloprevotella*, *Porphyromonas*, and *Neisseria* has been associated with increased disease severity, whereas *Veillonella* appears to play a protective role [93]. *Alloprevotella* has been proposed to contribute to inflammatory processes through activation of Toll-like receptor 2 (TLR2)-dependent signaling and subsequent NF-κB activation, leading to increased production of IL-23 and IL-1 and downstream expansion of Th17 lymphocytes, which play a central pathogenic role in psoriasis [60]. *Porphyromonas* species have been reported to produce arginine-rich peptides and proteolytic enzymes that may promote protein citrullination, disrupt epithelial barrier integrity, and enhance local cytokine release, thereby intensifying inflammatory processes, similarly to mechanisms described in rheumatoid arthritis [60]. Neisseria may promote inflammation by enhancing antigen-presenting cell activation, upregulating costimulatory molecules, and increasing downstream production of inflammatory mediators and effector T-cell activation [60]. *Veillonella*, on the other hand, was negatively correlated with psoriasis severity, suggesting a potential protective role. This bacterium is known to produce propionic acid, a short-chain fatty acid with anti-inflammatory properties, which suppresses NF-κB signaling and reduces the production of key psoriasis-related cytokines, including IL-17 and TNF-α [60]. Reduced levels of *Veillonella* in psoriasis patients may be associated with lower propionic acid production, which could weaken anti-inflammatory responses and potentially contribute to chronic skin inflammation. In addition to bacterial shifts, culture-based studies summarized in a recent systematic review showed an increased prevalence and load of oral *Candida* spp. in patients with psoriasis compared with healthy controls. In some cohorts, this colonization correlated with disease severity, potentially through enhancement of IL-17-driven mucosal immune responses and chronic activation of antifungal Th17 immunity [96].

*Prevotella* and *Porphyromonas gingivalis* have been proposed to activate TLR2 signaling pathways, which may enhance the production of IL-23 and IL-1β involved in inflammatory responses associated with psoriasis [60]. Meanwhile, the decreased abundance of *Haemophilus*, also observed in rheumatoid arthritis, may impair the microbiota’s ability to regulate immune responses and contribute to chronic inflammation [98]. Previous studies have shown that alterations in skin and gut microbiota play a central role in inflammatory skin diseases [99,100]. This concept is further supported by recent systematic reviews of the gut microbiome in psoriasis, which consistently describe intestinal dysbiosis, altered *Firmicutes*/*Bacteroidetes* ratios, and significant differences in β-diversity between patients and controls, reinforcing the hypothesis of a gut–skin axis and microbiota-driven immune modulation in psoriasis through short-chain fatty acid-dependent regulation of Treg/Th17 balance and systemic cytokine signaling [101].

LEfSe analysis revealed that the saliva of psoriasis patients was significantly enriched in *Bacteroidetes*, *Patescibacteria*, *Prevotellaceae*, *Porphyromonadaceae*, *Alloprevotella*, *Porphyromonas,* and *Prevotella*_7, indicating their potential involvement in promoting inflammatory responses through activation of pro-inflammatory pathways such as the Th17 axis and cytokine production (including IL-23 and IL-1) [60]. In contrast, healthy individuals had saliva dominated by *Proteobacteria* and *Haemophilus*, indicating a more balanced microbiota and potentially better immune regulation [60]. These findings support the hypothesis that oral microbiota dysbiosis may be associated with heightened inflammatory responses and disease progression in psoriasis.

Nevertheless, both oral and gut microbiome studies in psoriasis are limited by small sample sizes, heterogeneous inclusion criteria, and differences in sampling procedures, sequencing platforms, and bioinformatic pipelines. These limitations currently preclude the definition of a psoriasis-specific microbial signature and highlight the need for larger, methodologically standardized studies to precisely define causal microbe–host immune interactions driving psoriasis-associated oral and intestinal dysbiosis [96,101].

## 5. Limitations and Future Perspectives

Potential confounding factors should be considered when interpreting the results of studies assessing salivary biomarkers in psoriasis. Medications commonly used in psoriasis management, including systemic therapies and biologic agents, can influence immune responses, oxidative stress parameters, and salivary gland function. Dietary habits and nutritional status can modulate antioxidant capacity and inflammatory markers, while oral hygiene practices and periodontal status may directly affect salivary cytokine levels, oxidative stress markers, and oral microbiota composition. Additionally, comorbidities frequently associated with psoriasis, such as metabolic syndrome, diabetes mellitus, cardiovascular diseases, and other autoimmune disorders, may independently alter salivary biomarker profiles. Furthermore, substantial heterogeneity exists in saliva collection, processing, and storage methods across the included studies. Differences regarding the type of saliva collected (non-stimulated versus stimulated), time of sampling, and fasting status may significantly affect biomarker stability and measured concentrations. Additional sources of variability include pre-sampling restrictions (food intake, smoking, oral hygiene), centrifugation protocols, storage temperature, and storage duration. The lack of uniform control for these confounding and methodological variables across the included studies represents an important source of heterogeneity and must be taken into account when interpreting the reported findings. Future studies should aim to standardize participant selection, saliva collection, and processing protocols to improve the reliability and clinical applicability of salivary biomarkers in psoriasis.

Future research should focus on large-scale, multicenter, and longitudinal studies with standardized saliva collection and processing protocols. The inclusion of well-defined clinical phenotypes, detailed medication profiles, and rigorous control of confounding factors will be essential to improve data comparability. Moreover, the integration of salivary biomarkers with clinical indices, imaging data, and other biological matrices may help establish their diagnostic, prognostic, and therapeutic monitoring value in psoriasis.

Recent machine learning-based transcriptomic studies in psoriatic skin have identified novel diagnostic gene signatures and dynamic patterns of immune cell infiltration. These findings illustrate how omics-driven approaches could be combined in the future with salivary biomarker profiling to develop integrated, multi-parameter diagnostic and monitoring tools in psoriasis [102].

## 6. Conclusions

Current evidence suggests a potential role of salivary biomarkers in the pathogenesis and clinical monitoring of psoriasis. Elevated salivary amylase activity and alterations in sIgA and lysozyme levels may be associated with impaired innate immunity in the oral cavity of patients with psoriasis, which may increase susceptibility to infections. An increase in TOS and OSI appears to be associated with the severity of skin lesions, supporting the potential involvement of oxidative stress in disease mechanisms. Disturbances in the salivary cytokine profile, including elevated levels of TNF-α, IL-2, and IFN-γ and reduced IL-10, may reflect a chronic inflammatory state and a broader pro-inflammatory immune milieu, including Th1-related signals within the broader IL-23/Th17-driven immunopathology of psoriasis. Biological therapy has been associated with beneficial changes in salivary biomarker levels, suggesting a potential immunomodulatory effect, although the available evidence remains limited. Alterations in the oral microbiota, including increased abundance of *Alloprevotella* and *Porphyromonas*, may be associated with inflammatory processes; however, current evidence is limited and does not allow causal conclusions. Salivary biomarkers such as amylase, sIgA, TNF-α, and oxidative stress parameters may serve as potential non-invasive indicators of disease activity and treatment response in psoriasis; however, their clinical utility requires further validation in large-scale, standardized, and longitudinal studies.

## Figures and Tables

**Table 1:** Inclusion and exclusion criteria according to the PECOS framework.

Parameter	Inclusion Criteria	Exclusion Criteria
Population	Results of studies conducted on humans. Patients aged from 0 to 99 years, both genders	Results of studies without human participants (e.g., studies on animal models or in vitro).
Exposure	Psoriasis	Other skin diseases (e.g., bullous diseases, atopic dermatitis), psoriasis coexisting with other diseases, e.g., periodontitis
Comparison	Not applicable	
Outcomes	Publications regarding the use of saliva as a diagnostic material for the assessment of oxidative stress and inflammation in patients with psoriasis.	Publications not including the use of saliva as a diagnostic material for the assessment of oxidative stress and inflammation in patients with psoriasis.
Study Design	Original research articles, pilot studies, and research letters containing original data in English	Literature reviews, case reports, commentaries and others in a language other than English

**Table 2:** Quality assessment, including the main potential risk of bias.

	Clearly Stated Research Question or Objective	Clearly Defined Study Population	Sample Size Justification	Groups Recruitment from the Same Population	Valid Inclusion and Exclusion Criteria	Cases Differentiated from Controls	Randomization	Clearly Defined Measures	Blinded Participant Status	Adjusted Statistical Methods	Summary Quality Score
Zhao 2024 [60]	🟢	🟢	🔴	🟡	🟢	🟢	🔴	🟢	🔴	🟡	🟡
Sharma, 2024 [61,62]	🟢	🟢	🟡	🟢	🟢	🟢	🔴	🟢	🔴	🟢	🟢
Sharma, 2023 [63]	🟢	🟢	🟡	🟢	🟢	🟢	🔴	🟢	🔴	🟢	🟢
Foks-Ciekalska, 2023 [64]	🟢	🟢	🟡	🟢	🟢	🟢	🟡	🟢	🔴	🟢	🟢
Repousi, 2022 [65]	🟢	🟢	🔴	🟢	🟢	🟢	🔴	🟢	🔴	🟢	🟡
Skutnik-Radziszewska, 2020 [42]	🟢	🟢	🟡	🟢	🟢	🟢	🔴	🟢	🔴	🟢	🟢
Skutnik-Radziszewska, 2020 [41]	🟢	🟢	🟡	🟢	🟢	🟢	🔴	🟢	🔴	🟢	🟢
Ganzetti, 2016 [66]	🟢	🟢	🔴	🟢	🟢	🟢	🔴	🟢	🔴	🟢	🟡
Soudan, 2011 [67]	🟢	🟢	🔴	🟢	🟢	🟢	🔴	🟢	🔴	🟢	🟡
Mastrolonardo, 2007 [68]	🟢	🟢	🟡	🟢	🟢	🟢	🔴	🟢	🔴	🟢	🟢
Koh 2004, [69]	🟢	🟢	🟡	🟢	🟢	🟢	🔴	🟢	🔴	🟢	🟢

risk level: green—low risk, yellow—unclear risk, red—high risk; quality score: green—good, yellow—intermediate, red—poor [41,42,60,61,62,63,64,65,66,67,68,69].

**Table 3:** General characteristics of included studies.

Author, Year	Type of Study	Analyzed Parameters and Molecules	Sample Type	Method of Analysis	Inclusion and Exclusion Criteria	Number of Patients	Results
Zhao, 2024 [60]	case–control study	oral microbiota variations	nonstimulated whole saliva (NWS)	DNA extraction: TGuide S96 Magnetic Soil/Stool DNA Kit Tiangen Biotech (Beijiing, China). 16S rRNA sequencing: Primers 27F/1492R, PCR (25 cycles), PacBio Sequel II platform. Bioinformatics: Sequence filtering and assignment (SMRT Link v8.0, lima v1.7.0), OTU clustering (USEARCH v10.0), taxonomy (SILVA v138, Naive Bayes Classifier, BLAST). Diversity analysis: Alpha: Shannon Index, Gini-Simpson Index Beta: PCoA, NMDS Statistical analysis: Wilcoxon test, Spearman correlation, LEfSe (LDA score > 4.0), NWS volume: 1 mL	Inclusion criteria: Clinically confirmed plaque psoriasis (diagnosed by two dermatologists); age 18–60; Han Chinese population. Exclusion criteria: Use of antibiotics, probiotics, corticosteroids, or immunosuppressants within the past 3 months; pregnancy or breastfeeding; presence of autoimmune, metabolic, neurological, psychiatric, or infectious diseases.	n = 20 (13 males (M), 7 females (F))—psoriasis group n = 20 (12 M, 8 F)—healthy controls	Alpha diversity of the oral microbiota was higher in psoriasis patients (*p* < 0.05, Gini-Simpson index). Beta diversity showed significant differences in microbial composition between groups (*p* < 0.05). Increased abundance of *Prevotella*, *Prevotella*_7, *Porphyromonas*, and decreased *Haemophilus* in the psoriasis group (*p* < 0.05). Positive correlation of *Alloprevotella*, *Porphyromonas*, *Neisseria* with psoriasis severity (PASI, PGA, BSA); negative correlation with *Veillonella* (*p* < 0.05). LEfSe analysis: psoriasis patients were enriched in *Bacteroidetes*, *Patescibacteriota*, *Prevotellaceae*, *Porphyromonadaceae*, *Alloprevotella*, *Porphyromonas*, *Prevotella*_7, *Prevotella melaninogenica*; healthy controls were dominated by *Proteobacteria* and *Haemophilus* (*p* < 0.05).
Sharma, 2024 [61,62]	research letter, case–control study	Tumor Necrosis Factor-alpha (TNF-α), Interferon-gamma (INF-γ), Interleukin-2 (IL-2), Interleukin-10 (IL-10).	NWS	Cytokine measurement: ELISA for TNF-α, IFN-γ, IL-2, IL-10 Salivary flow analysis: Volume measured in mL/min Statistical analysis: Spearman correlation, Student’s *t*-test (*p* < 0.05 considered significant) NWS collection time: 5 min	Inclusion criteria: Clinically confirmed plaque psoriasis; age 21–68 years. Exclusion criteria: Use of immunosuppressive drugs; presence of autoimmune, metabolic, or neurological disorders.	n = 60 (46M, 14F)—psoriasis group, including: n = 33 (24M, 9F)—mild to moderate psoriasis n = 27 (22M, 5F)—severe psoriasis n = 60 (47M, 13F)—healthy controls	↑ TNF-α, IFN-γ, IL-2 in NWS of psoriasis patients vs. healthy controls ↓ IL-10 in NWS of psoriasis patients vs. healthy controls ↑ TNF-α in NWS of patients with mild to moderate psoriasis vs. healthy controls ↑ TNF-α, IFN-γ, IL-2 in NWS of patients with severe psoriasis vs. healthy controls ↑ IFN-γ, IL-2 in NWS of patients with severe vs. mild to moderate psoriasis ↓ IL-10 in NWS of patients with severe psoriasis vs. healthy controls
Sharma, 2023 [63]	case–control study	TNF-α IFN-γ IL-2 IL-10	NWS	Salivary cytokines: ELISA for TNF-α, IFN-γ, IL-2, IL-10 Salivary flow rate: Measured in mL/min Statistical analysis: Mann–Whitney U test, ANOVA, Kruskal–Wallis test, Pearson correlation (*p* < 0.05) NWS collection time: 5 min	Inclusion criteria: Plaque psoriasis; age 21–68 years; no treatment within the past month. Exclusion criteria: Unstable psoriasis; autoimmune diseases; oral diseases; alcoholism; hormone replacement therapy (HRT); disorders of the endocrine, hepatic, or renal systems; pregnancy/lactation.	n = 60 (46M, 14F)—psoriasis group, including: n = 28 (22M, 6F)—psoriasis with normal salivation n = 32 (24M, 8F)—psoriasis with hyposalivation n = 60 (47M, 13F)—healthy controls	↑ TNF-α, IL-2 in NWS of patients with psoriasis and normal salivation vs. healthy controls ↑ TNF-α, IFN-γ, IL-2 in NWS of patients with hyposalivation vs. healthy controls ↓ IL-10 in NWS of patients with psoriasis and normal salivation vs. healthy controls ↓ IL-10 in NWS of patients with hyposalivation vs. healthy controls
Foks-Ciekalska, 2023 [64]	case–control study	α-amylase (sAA) saliva concentration of immunoglobulin A (sIgA), chromogranin A (CgA)	NWS	sAA: Spectrophotometry (Aqua-Med, Lodz, Poland), sIgA: ELISA Immunodiagnostic AG (Bensheim, Germany), CgA: ELISA Cisbio Bioassays (Codolet, France), Statistical analysis: Friedman test, Conover post hoc test (*p* < 0.05) NWS volume: 3 mL	Inclusion criteria: Age >18 years; severe psoriasis defined as Psoriasis Area and Severity Index (PASI) > 18, Dermatology Life Quality Index (DLQI) > 10, and Body Surface Area (BSA) > 10; no prior biological treatment; stable systemic health condition allowing participation in the study; signed informed consent. Exclusion criteria: Pregnancy or lactation; history of malignant neoplasms (except successfully treated basal cell carcinoma); chronic infections including human immunodeficiency virus (HIV), hepatitis B virus (HBV), and active tuberculosis; systemic or autoimmune diseases such as systemic lupus erythematosus (SLE) or severe inflammatory rheumatic conditions; severe heart failure (New York Heart Association (NYHA) class III/IV); neurological disorders suggestive of central nervous system (CNS) demyelination; use of immunosuppressive drugs within the past 3 months.	n = 84 (53M, 29F)—severe psoriasis, biological treatment n = 20 (12M, 8F)—severe psoriasis, symptomatic treatment	↑ sAA in NWS of patients with severe psoriasis undergoing biological treatment as therapy progressed ↑ sIgA in NWS of patients with severe psoriasis undergoing biological treatment as therapy progressed ↓ CgA in NWS of patients with severe psoriasis undergoing biological treatment as therapy progressed
Repousi, 2022 [65]	case–control study	cortisol	NWS	Salivary cortisol measurement: Electrochemiluminescence immunoassay (ECLIA, Cobas e411, Roche Diagnostics) Statistical analysis: Student’s *t*-test, Pearson correlation, multivariate regression (*p* < 0.05) Sampling schedule: 5 NWS samples collected throughout the day (08:00, 12:00, 15:00, 18:00, 21:00).	Inclusion criteria: Age 18–65 years; flare-up of non-pustular psoriasis diagnosed by a dermatologist. Exclusion criteria: Acute infectious disease; severe chronic conditions (heart, liver, or kidney failure); long-term corticosteroid use (>1 month); neurological or psychiatric disorders.	n = 18 (16M, 2F)—psoriasis group n = 18 (16M, 2F)—healthy controls	Psoriasis patients showed a disrupted diurnal rhythm of cortisol concentration in NWS. Positive correlation between cortisol levels in NWS and anxiety severity. Positive correlation between cortisol levels in NWS and PASI score.
Skutnik-Radziszewska, 2020 [42]	case–control study	peroxidase (Px), catalase (CAT), superoxide dismutase (SOD), total antioxidant capacity (TAC), total oxidative status (TOS), oxidative stress index (OSI), reactive oxygen species (ROS) production, advanced glycation end products (AGE), advanced oxidation protein products (AOPP), malondialdehyde (MDA), total lipid hydroperoxides (LOOH).	NWS, stimulated whole saliva (SWS)	Antioxidants: Px, CAT, SOD—spectrophotometry Oxidative stress: TOS—spectrophotometry Oxidative modifications: AGE, AOPP, MDA, LOOH—ELISA Statistical analysis: Student’s *t*-test, Mann–Whitney U test, Pearson correlation, ROC analysis (*p* < 0.05) $OSI=\frac{TOS}{TAC}$ Sample collection time: NWS—10 min, SWS—5 min.	Inclusion criteria: Plaque psoriasis; symptom exacerbation; no systemic treatment for at least 2 years; absence of systemic diseases affecting oxidative stress. Exclusion criteria: Autoimmune diseases; metabolic disorders (e.g., diabetes, gout, osteoporosis); infectious diseases (HIV, HBV, Hepatitis C Virus [HCV]); oral diseases (periodontitis, pathological lesions of the oral mucosa); use of medications including nonsteroidal anti-inflammatory drugs (NSAIDs), glucocorticosteroids, or antibiotics within the past 6 months.	n = 40 (27M, 13F)—psoriasis n = 40 (27M, 13F)—healthy controls	↑ Px, SOD, TOS, OSI, AGE, AOPP, ROS production, MDA, and LOOH in NWS and SWS of psoriasis patients vs. healthy controls, ↑ CAT in NWS of psoriasis patients vs. healthy controls, ↑ TAC in SWS of psoriasis patients vs. healthy controls
Skutnik-Radziszewska, 2020 [41]	case–control study	TNF-α IFN-γ IL-2 IL-10	NWS SWS	Salivary cytokines: ELISA for TNF-α, IL-2, IFN-γ, IL-10 (EIAab Science Inc., Wuhan, China) Statistical analysis: ANOVA, Tukey’s test, Pearson correlation, ROC (*p* < 0.05) Sample collection time: NWS—15 min; SWS—5 min	Inclusion criteria: Plaque psoriasis; symptom exacerbation; no systemic treatment for at least 2 years; absence of systemic diseases affecting oxidative or nitrosative stress; no periodontal or oral mucosal diseases; probing pocket depth (PPD) < 2 mm; no bleeding on probing. Exclusion criteria: Autoimmune diseases; metabolic disorders (e.g., diabetes, gout, osteoporosis); infectious diseases (HIV, HBV, HCV); neurological or psychiatric disorders; use of medications including NSAIDs, corticosteroids, or antibiotics within the last 6 months; smoking, alcoholism, or acrylic dentures.	n = 30 (13M, 17F)—psoriasis with normal salivation (PN) n = 30 (10M, 20F)—psoriasis with hyposalivation (PH) n = 60 (23M, 37F)—healthy controls (C)	↑ TNF-α, IFN-γ, IL-2 in NWS in PH vs. PN, ↑ TNF-α, IFN-γ, IL-2 in NWS in PH vs. C, ↑ IFN-γ, IL-2 in NWS in PN vs. C, ↑ TNF-α, IFN-γ, IL-2 in SWS in PH vs. PN ↑ TNF-α, IFN-γ, IL-2 in SWS in PH vs. C ↑ TNF-α, IL-2 in SWS in PN vs. C ↓ IL-10 in NWS in PH vs. PN ↓ IL-10 in NWS in PH vs. C ↓ IL-10 in NWS in PN vs. C ↓ IL-10 in SWS in PH vs. PN ↓ IL-10 in SWS in PH vs. C ↓ IL-10 in SWS in PN vs. C
Ganzetti, 2016 [66]	pilot study	Interleukin 1 Beta (IL-1β)	NWS	IL-1β: ELISA (Qiagen, Venlo, The Netherlands). Psoriasis severity assessment: PASI. Statistical analysis: Kruskal–Wallis test, linear regression, GraphPad Prism 5 (*p* < 0.05). Sample collection: NWS collected from both the control and study groups; in the study group, a follow-up NWS sample was collected after 12 weeks.	Inclusion criteria: Stable plaque psoriasis, no periodontal disease, no prior biological treatment. Exclusion criteria: Periodontal and oral diseases, history of treatment with TNF-α inhibitors.	n = 25 (15M, 10F)—psoriasis, treated with TNF-α inhibitor (biological therapy) n = 20 (12M, 8F)—healthy controls	↑ IL-1β levels in psoriasis patients vs. control group. Treatment with TNF-α inhibitors led to ↓ IL-1β levels in NWS of psoriasis patients; however, levels remained higher than in the control group.
Soudan, 2011 [67]	case–control study	sAA	NWS	sAA: Measured via ELISA 500 Plus (Biomerieux) after 1:200 dilution Salivary pH: Assessed using colorimetric pH indicators Statistical analysis: Student’s *t*-test, Pearson’s correlation, SPSS 13.0 (*p* < 0.05) Collection: NWS collected by spitting method for 5 min	Inclusion criteria: Plaque psoriasis, without systemic complications, no systemic treatment for at least 3 months. Exclusion criteria: Systemic diseases including diabetes, hypertension, metabolic disorders, oral diseases including periodontitis, immunosuppressive treatment within the last 3 months.	n = 20 (10M, 10F)—psoriasis n = 20 (10M, 10F)—healthy controls	↑ sAA in NWS of patients with psoriasis vs. control.
Mastrolonardo, 2007 [68]	case–control study	IL-1β	SWS	IL-1β: ELISA (Euroclone Ltd., UK). Statistical analysis: Student’s *t*-test, repeated measures ANOVA, Pearson correlation (*p* < 0.05). SWS collection: Using a Salivette device (Sarstedt, Germany), (chewing a swab for 30–45 s).	Inclusion criteria: Age 18–65 years, plaque psoriasis of mild to moderate severity (PASI = 17.6 ± 3.1), no use of topical/systemic steroids in the last 3 months, no other chronic diseases or psychiatric disorders. Exclusion criteria: Psychiatric disorders (including substance abuse), steroid treatment within the last 3 months, presence of other chronic diseases.	n = 25 (11M, 14F)—psoriasis, n = 50 (22M, 28F)—healthy controls.	↑ IL-1β in SWS of individuals with psoriasis vs. control.
Koh 2004, [69]	case–control study	sIgA lysozyme	NWS	sIgA: ELISA Salimetrics HS-IgA kit, (Salimetrics LLC, State College, PA, USA). Lysozyme: ELISA. Statistical analysis: Student’s *t*-test, linear models controlling for age (*p* < 0.05 considered statistically significant). NWS collected via drooling method for 5 min.	Inclusion criteria: Age ≥ 18 years, plaque psoriasis of varying severity (assessed using PASI), no immunosuppressive treatment in the last 4 weeks. Exclusion criteria: Acute respiratory infection in the past week, use of systemic steroids or immunosuppressive drugs in the last 4 weeks, presence of other chronic skin conditions.	n = 51 (51M)—psoriasis, including: n = 24 (24M)—mild psoriasis, n = 27 (27M)—severe psoriasis, n = 24 (24M)—healthy controls.	↓ IgA in NWS of individuals with psoriasis vs. control, ↓ lysozyme in NWS of individuals with psoriasis vs. control.

Abbreviations: AGE (advanced glycation end products), AOPP (advanced oxidation protein products), BSA (body surface area), CAT (catalase), CgA (chromogranin A), CNS (central nervous system), DLQI (Dermatology Life Quality Index), F (female), HBV (Hepatitis B Virus), HCV (Hepatitis C Virus), HIV (Human Immunodeficiency Virus), HRT (hormone replacement therapy), IL-1β (interleukin 1 beta), IL-2 (interleukin 2), IL-4 (interleukin 4), IL-6 (interleukin 6), IL-8 (interleukin 8), IL-10 (interleukin 10), IL-12 (interleukin 12), IL-17A (interleukin 17A), INF-γ (interferon-gamma), IgA (immunoglobulin A), LOOH (total lipid hydroperoxides), M (male), MDA (malondialdehyde), NSAID (nonsteroidal anti-inflammatory drugs), NWS (nonstimulated whole saliva), NYHA (New York Heart Association), OSI (oxidative stress index), PASI (Psoriasis Area and Severity Index), PGA (Physician Global Assessment), PH (psoriasis patients with hyposalivation), PN (psoriasis patients with normal salivation), PPD (probing pocket depth), Px (peroxidase), ROS (reactive oxygen species), sAA (α-amylase), sIgA (saliva concentration of immunoglobulin A), SLE (systemic lupus erythematosus), SOD (superoxide dismutase), SWS (stimulated whole saliva), TAC (total antioxidant capacity), TNF-α (tumor necrosis factor-alpha), TOS (total oxidative status).

## Data Availability

All data supporting the findings of this study are available within the article.

## Supplementary Materials

The following supporting information can be downloaded at: https://www.mdpi.com/article/10.3390/dj14030184/s1, Supplementary Table S1: Pre-analytical characteristics of saliva sampling protocols in the included studies; PRISMA Checklist.
