# Supplementary material for: Changes in Salivary Biomarkers and Oral Immune Parameters in Patients with Psoriasis: A Systematic Review
Source: Dent J (Basel). 2026 Mar 19;14(3):184. doi: 10.3390/dj14030184 (PMC13025884; doi:10.3390/dj14030184)
Supplement: Supplementary file 1 [file dentistry-14-00184-s001.zip › dentistry-4162083-supplementary.pdf]

Table S1. Pre-analytical characteristics of saliva sampling protocols in the included studies. NWS – non-stimulated whole saliva; SWS – stimulated whole saliva.

Study	Saliva type	Collection duration	Sampling time	Pre-sampling restrictions	Analytical method
Zhao 2024 [97]	NWS	~1 mL saliva	not specified	antibiotics/probiotics excluded	16S rRNA sequencing
Sharma 2024 [71,72]	NWS	5 min	09:00-11:00	not specified	ELISA
Sharma 2023 [73]	NWS	5 min	not specified	no treatment within 1 month	ELISA
Foks-Ciekalska 2023 [74]	NWS	~3 mL saliva	not specified	no biologic therapy before study	spectrophotometry / ELISA
Repousi 2022 [60]	NWS	multiple samples collected	08:00-21:00	not specified	Electrochemiluminescence immunoassay
Skutnik-Radziszewska 2020 [42]	NWS + SWS	10 min / 5 min	08:00-10:00	no systemic treatment	spectrophotometry / ELISA
Skutnik-Radziszewska 2020 [41]	NWS + SWS	15 min / 5 min	not specified	no systemic treatment	ELISA
Ganzetti 2016 [61]	NWS	not specified	09:00-11:00	no periodontal disease	ELISA
Soudan 2011 [65]	NWS	5 min	not specified	no systemic treatment	ELISA
Mastrolonardo 2007 [66]	SWS	~30–45 s	14:30-15:30	no steroid therapy	ELISA
Koh 2004 [67]	NWS	5 min	not specified	no immunosuppressive therapy	ELISA
